# Results of routine thoracic radiographic examinations rarely impact the plan to proceed with anesthetic procedures in dogs with presumed acute intervertebral disc herniation

**DOI:** 10.3389/fvets.2024.1385814

**Published:** 2024-04-08

**Authors:** Teiko Takedai, Nicole Block, Karina M. Pinal, Steven W. Frederick

**Affiliations:** ^1^Surgery Department, BluePearl Specialty and Emergency Pet Hospital, Maitland, FL, United States; ^2^Neurology Department, BluePearl Specialty and Emergency Pet Hospital, Sandy Springs, GA, United States; ^3^Science, BluePearl Specialty and Emergency Pet Hospital, Sandy Springs, GA, United States

**Keywords:** IVDH, thoracic radiographs, imaging, ALARA, dog

## Abstract

**Introduction:**

Acute thoracolumbar intervertebral disc herniation (IVDH) constitutes an emergency because associated neurological clinical signs can be progressive, with prognosis dependent on preoperative presence of deep pain perception. Pre-anesthetic thoracic radiographs are routinely performed to evaluate for potential pathology that could result in increased risk or change in overall prognosis. However, due to the emergent nature of this disease, the weight of thoracic radiographic findings on treatment plans for these dogs in unknown. The objective of this study was to investigate the clinical benefit of thoracic radiographs prior to advanced spinal imaging and surgery for acute non-ambulatory dogs with suspected T3-L3 myelopathy.

**Methods:**

Consecutive dogs presented with presumed acute IVDH between July 2020-July 2022 were identified, and medical records were reviewed retrospectively. Age, body weight, and alteration of treatment plan were compared between the dogs with positive thoracic pathology identified on thoracic radiographs and those with negative thoracic pathology.

**Results:**

Only 2/105 dogs had their diagnostic and treatment plans changed due to thoracic radiographic findings. Dogs with radiographic pathology diagnosed (2/16) were more likely to have their treatment plans changed than dogs with no radiographic pathology (0/89) (*p* = 0.014). The odds of radiographic thoracic pathology were 4.6 times higher in dogs aged 12 years or older [OR 4.6 (95%CI 1.2–17, *p* = 0.026].

**Discussion:**

Performing routine thoracic radiography prior to advanced (anesthetized) diagnostic spinal imaging in presumed IVDH cases rarely resulted in a change to the treatment plan, thought eh practice may be clinically relevant in dogs 12 years of age or older.

## 1 Introduction

Intervertebral disc herniation (IVDH) is a common neurologic disease of the vertebral column and spinal cord segments (1) that is categorized as compressive (Hansen type I, acute compressive nucleus pulposus extrusion; Hansen type II, annulus fibrosus protrusion) and non-compressive [type III, which is more recently referred to as acute non-compressive nucleus pulposus extrusion (ANNPE)] (2). Depending on severity of clinical signs, compressive IVDH may be treated surgically or medically, while non-compressive IVDH is treated medically (3). Due to the similarities in clinical signs and neurological examination findings in all types of acute IVDH, it is difficult to definitively diagnose the type of herniation without advanced imaging and/or surgical visualization of the disc material in the spinal canal, so the broad term IVDH is often used until a definitive diagnosis has been made. Acute IVDH most commonly causes compression of the spinal cord in the thoracolumbar segment (T3-L3), and dogs with IVDH affecting the thoracolumbar spinal cord are often presented with some degree of pelvic limb dysfunction ranging from ataxia to non-ambulatory paraparesis or paraplegia (4). Prolonged compression of the spinal cord due to IVDH may result in persistent clinical signs, and as a result, acute, severe disc-associated spinal cord injury (presumed IVDH) represents a time-sensitive, emergent disease often requiring immediate diagnosis with advanced imaging and treatment with surgical decompression (4–6).

In veterinary medicine, magnetic resonance imaging (MRI) examinations are performed under general anesthesia to prevent patient movement during image acquisition, and general anesthesia is not without risk (7). Prior to any anesthetic procedure, diagnostic screening tests including auscultation of the heart and lungs and preliminary biochemistry and hematology are often performed to determine if the dog is a good anesthetic candidate, with the expectation that diagnostic findings can be medically managed prior to the anesthetic procedure (8). Additionally, clinicians may recommend thoracic radiography in presumed IVDH cases as a preanesthetic screening procedure to evaluate for potential pathology that could result in increased anesthetic risk or change in overall prognosis (9).

Radiography is accessible, reliable, and relatively quick as a diagnostic tool in veterinary medicine, making it a great option for routine screening (9). However, radiography is not without risk. Exposure to ionizing radiation presents a risk to patients and healthcare workers, especially due to its cumulative effect, prompting the recommendation from the Centers for Disease Control to minimize radiation exposure through the “as low as reasonably achievable” (ALARA) principle (10–12). Additionally, in veterinary medicine either chemical restraint (risk to patients) or manual restraint (risk to patients and healthcare workers) must be used during radiographic positioning for diagnostic images (12, 13). These healthcare risks and the financial costs associated with radiography indicate that radiography as a screening tool should be reassessed.

However, while radiography is a useful diagnostic tool, it carries additional financial and safety costs for clients, patients, and healthcare workers, and due to IVDH’s emergent status, it is unclear if diagnostic findings on the thoracic radiographic examinations would impact treatment recommendations (12, 13). The objective of this study was to assess the clinical benefit of performing routine thoracic radiographic examinations prior to advanced spinal imaging and subsequent spinal decompression surgery for dogs with acute T3-L3 myelopathy. We hypothesized that the results of routine preanesthetic thoracic radiographs would be the cause of changed treatment plan recommendations in less than 5% of dogs presented for acute T3-L3 myelopathy.

## 2 Materials and methods

Medical records from the neurology service at a veterinary specialty and emergency hospital were reviewed to identify all dogs presented within 48 h of developing clinical signs of non-ambulatory T3-L3 myelopathy and diagnosed with presumed IVDH based on physical and neurological examination findings between July 2020 and July 2022. Dogs were included if they underwent neurologic evaluation by a board-certified veterinary neurologist or neurology resident under supervision of a board-certified veterinary neurologist, were recommended to have advanced spinal diagnostic imaging examination, had routine thoracic radiographs performed as part of their preanesthetic screening protocol (standard departmental procedure), had the radiographic examination findings reported by a board-certified veterinary radiologist, and either had the planned advanced spinal imaging performed or had medical record notes detailing the reason that the planned advanced spinal imaging was not performed. Routine radiographic examination was defined as radiographic examinations performed strictly due to protocol rather than being prompted by existing disease or physical examination findings (e.g., abnormal lung sounds). Dogs were excluded if the findings of the physical and neurological examination were inconsistent with T3-L3 myelopathy, thoracic pathology (e.g., pneumonia) was suspected based on auscultation prior to the thoracic radiography, or if the medical record notes indicated that due to the severe neurologic status or presumed poor prognosis following initial physical and neurological examination, the dog was not recommended to pursue advanced spinal imaging.

Information collected from medical records included signalment, body weight, neurologic examination results, neurologic status at presentation, thoracic radiographic examination report findings, and all medical notes discussing treatment plans. Treatment plans were evaluated via examination and client communication notes in the medical records at the time of initial presentation, after the physical and neurologic examination, and after the addition of the radiologist’s report about the thoracic radiographic examination. A change in treatment plan was documented when either the clinician documented a deviation from the previous recommendation, or the medical records indicated that the client elected to postpone or decline advanced diagnostic imaging as a result of the thoracic radiographic examination report.

All analyses were performed using SAS 9.4 (Cary, NC). A significance threshold of *p* ≤ 0.05 was used. An assumption of normality was evaluated via inspection of QQ-plots, histograms, and skewness. All variables were summarized descriptively with median, interquartile range and minimum and maximum and mean and standard deviations were additionally provided for normally distributed variables (age). Two-sided Wilson confidence intervals were calculated for binomial proportions. Fisher’s exact tests were used to test for associations of having a thoracic pathology with plans changing and having anesthetic imaging done. Logistic regressions were used to estimate odds-ratios and test for the effects of breed, age, and weight on having a thoracic pathology. Breeds with less than 5 animals were grouped into an “other” category. Age was analyzed both as a continuous variable and a categorical variable with a cut-point of greater than or equal to 12 years.

## 3 Results

A total of 105 dogs met all inclusion criteria. There were 67 male dogs (63 neutered, 4 intact) and 38 female dogs (33 spayed, 5 intact). Twenty-seven American Kennel Club breeds represented 70 of the dogs, and the remaining 35 dogs were mixed breed dogs. Dachshunds (*n* = 35) and French Bulldogs (*n* = 8) were the only breeds with more than 3 participants, and the remaining breeds were grouped into the “other” category for analyses. Mean (± SD) age of all the dogs was 7.7 (± 3.2) years, and median (IQR) body weight was 9.5 (6.8–15.8) kg. Sixteen of 105 (15.2%) dogs had abnormal pathology diagnosed on routine thoracic radiographic examination. The abnormal pathologies diagnosed included cardiomegaly (*n* = 5), pneumonia (*n* = 3), mass (*n* = 3), and one each of bronchitis, non-spinal degenerative joint disease, hepatomegaly, fluid in the caudal esophagus, microhepatica, and hypovolemia. One dog was diagnosed with both cardiomegaly and lower airway disease.

Thirteen of 105 [12% (95%CI) 7–20%] dogs had abnormal pathology identified specifically in the thoracic cavity, and the remaining three abnormal findings were extra-thoracic (e.g., lytic skeletal lesion of the scapula). Two of 13 [15% (95%CI) 4–42%] of the dogs with abnormal pathology had their treatment plans changed, and none (0%) of the dogs with extra-thoracic radiographic diagnoses had their treatment plan changed. One of the two dogs that had their plan changed presented with severe paraparesis and had a small amount of fluid in the caudal thoracic esophagus. Therefore, medical therapy was recommended given it was a brachycephalic breed and this finding further increased anesthetic risk. The second case that had treatment altered had multiple skeletal lesions on thoracic radiographs which may have been associated with previous trauma, metabolic bone disease or neoplastic process such as multiple myeloma (14). Given this finding, the owners elected humane euthanasia for this dog. No dogs with normal radiographic thoracic evaluation had their treatment plans changed [0% (95%CI) 0–4%].

An anesthetic event for advanced spinal imaging was pursued in 11/16 [69% (95%CI) 44–86%] of dogs with abnormal thoracic pathology and 83/89 [93% (95%CI) 86–97%] of dogs with no diagnosis of thoracic pathology. Dogs with abnormal pathology were more likely to have their treatment plan changed (*p* = 0.014) and less likely to proceed with advanced spinal imaging (*p* = 0.029). No specific breed was at greater risk than another for having thoracic pathology identified on routine radiographic examination (*p* = 0.081) (Table 1). There was no significant association of odds of having a thoracic pathology and weight [per 5 lb, OR 1.2 (95% CI) 0.9–1.6, *p* = 0.142] or age [per 1 year, OR 1.2 (95%CI) 1.0–1.4, *p* = 0.056] when analyzed as continuous variables. However, dogs aged 12 years or older were at 4.6 times greater risk of having radiographically diagnosed thoracic pathology than dogs younger than 12 years old [OR 4.6 (95%CI) 1.2–17, *p* = 0.026].

**Table 1:** Risk factors for radiographic pathology.

Risk factor	Comparison	Odds ratio (95%CI)	*p*-value
Breed	Dachshund vs. Other	3.7 (0.4–77)	0.081
	French Bulldog vs. Other	4.6 (0.2–126)	
	Mixed vs. Other	9.5 (1.6–182)	
Weight	per 5 lb	1.2 (0.9–1.6)	0.142
Age	Per year	1.2 (1.0–1.4)	0.056
Age ≥ 12	≥12 vs. <12	4.6 (1.2–17)	0.026*

*Indicates significance.

## 4 Discussion

In this population, dogs presented with suspected acute IVDH were statistically more likely to have altered treatment recommendations when abnormal thoracic pathology was identified on routine thoracic radiographic screening examinations compared to dogs with no abnormal examination findings. However, while routine radiographic evaluation identified abnormalities in 16/105 (15.2%) of dogs, the results only prompted a change in treatment plan for 2/105 (0.02%) of total dogs. This is presumably due to the emergent nature of acute IVDH (or acute progression of chronic IVDH), and potentially poor outcomes associated with delayed diagnosis and treatment (4). Therefore, we accepted our hypothesis.

Literature describing the incidence of preoperative comorbidity in dogs presenting with acute paraparesis or paraplegia is scarce (15–21). Various risk factors (i.e., breed, body weight, medications) have been extensively reported regarding overall prognosis following surgical decompression as well as postoperative complications (15–21); however, lack of data exists regarding incidence of preoperative risk factors. Often, dogs with existing risk factors are excluded from these studies to standardize the data and to eliminate these variables as confounding factors. The incidence of pathology identified on radiographic screening examination in this study (15.2%) is similar to the 16% of dogs with comorbidities at the time of acute IVDH presentation reported by Moore et al. (19). Though, the risk factors most commonly reported by Moore et al. were obesity, orthopedic disease, and cardiac disease, which were not directly investigated or incorporated into our study protocol, and it’s likely that dogs with known risk factors would have increased in both studies if overall health and routine screening radiography were employed (19).

The value of routine radiographic examinations during uneventful tibial plateau leveling osteotomy (22, 23) and stabilization of medial patellar luxations (24) has been similarly assessed and reported. In all cases, results of routine, screening radiographic examination were found to have little clinical impact on the recovery recommendations in these common orthopedic conditions. However, those studies evaluated postoperative outcomes rather than screening for anesthetic risk prior to emergency anesthesia and surgery. Interestingly, unlike in those studies, routine pre-anesthetic radiographs were found to be beneficial in the senior population of dogs presenting with T3-L3 myelopathy secondary to presumed acute IVDH. This is thought to be due to thoracic radiography in presumed acute IVDH dogs being used as a screening tool prior to anesthetic events for advanced diagnostic imaging (either CT-myelogram or MRI) with or without decompressive surgery, neither of which is inexpensive or without risks. However, its use as a screening tool in these cases may be of limited value due to the risk of lasting neurologic dysfunction if the anesthetic events are postponed due to the findings which may explain why only 2/16 of the dogs diagnosed with abnormal radiographic pathology had their treatment plans altered.

There were several limitations to our study. First, dogs were included in this study even if they did not have definitive diagnosis of IVDH via advanced imaging or decompressive surgery. However, veterinary neurologists are trained to isolate and make presumed diagnoses of IVDH or T3-L3 myelopathy regularly, and requiring dogs to have definitive diagnosis would have resulted in exclusion of all dogs that had their plan changed to medical management due to their routine thoracic radiographic examination findings. As with any retrospective study, the accuracy of the data was dependent on the accuracy of the medical records available. It is also possible that treatment recommendation may have varied depending on the primary clinician, and the outcomes may have been biased depending on the clinician. For example, one dog had a soft tissue opacity in cranial mediastinum which could have been associated with lymphadenopathy, thymoma or thymic cyst. The dog presented as paraplegic with absent deep pain. In this case, an MRI was still recommended and pursued, which revealed multifocal spinal masses. In another case, a focal pneumonia was identified on thoracic radiographs, however, the dog presented as paraplegic with absent deep pain. Similarly, in this dog, advanced imaging was recommended and performed, followed by hemilaminectomy. Some clinicians may be more conservative in their approach, and it is possible they may have advised against pursuing MRI given the intra-thoracic pathology identified in these cases (i.e., altering the plan).

In conclusion, the results of this study suggested that routine thoracic radiographic screening examination in dogs presented with acute T3-L3 myelopathy rarely prompted a change in the prescribed diagnostic or treatment plan. However, the odds of finding a radiographic thoracic pathology were nearly five times higher for dogs aged 12 years or older, and routine radiographic examination in that population may be of increased clinical importance despite the risks associated with radiography. Further clinical studies are warranted to conclude whether this finding is universal and applicable to the general population.

## Data availability statement

The raw data supporting the conclusions of this article will be made available by the authors, without undue reservation.

## Author contributions

TT: Formal analysis, Writing – original draft, Writing – review & editing. NB: Data curation, Formal analysis, Writing – original draft, Writing – review & editing. KP: Data curation, Formal analysis, Writing – original draft, Writing – review & editing. SF: Conceptualization, Formal analysis, Writing – original draft, Writing – review & editing.
